# Associations between objectively and subjectively measured sleep outcomes among elementary school children in Rhode Island

**DOI:** 10.3389/fped.2025.1622943

**Published:** 2025-09-22

**Authors:** Aliana Rodriguez Acevedo, Diane Story, Lovisa Werner, David H. Barker, John E. McGeary, Shira I. Dunsiger, Diana S. Grigsby-Toussaint

**Affiliations:** ^1^ Center for Health Promotion and Health Equity, Department of Behavioral and Social Sciences, School of Public Health, Brown University, Providence, RI, United States; ^2^ Survey Research Center, School of Public Health, Brown University, Providence, RI, United States; ^3^ Department of Psychiatry and Human Behavior, Warren Alpert School of Medicine, Brown University, Providence, RI, United States; ^4^ Department of Psychiatry, Rhode Island Hospital, Providence, RI, United States

**Keywords:** actigraphy, accelerometry, pediatric sleep, pediatric sleep guidelines, parental perception of sleep, sleep in latino children

## Abstract

**Objective:**

Although sleep is essential for healthy child development, parents generally misconstrue definitions of sleep duration and sleep quality. This study examines differences between objective and parent-reported measures of sleep in children, with a particular focus on Latino and non-Latino groups. We hypothesized that significant discrepancies exist between objective and subjective sleep measures and differences in sleep outcomes between Latino and non-Latino subgroups.

**Methods:**

Children in grades one, two, and three in Rhode Island (*n* = 102; age range 6–10 years; 57.8% female) wore an accelerometer for seven days to objectively measure time in bed, sleep duration, wake after sleep onset, and sleep latency. Parents also reported perceived sleep outcomes, and whether they believed their child generally “sleeps the right amount”. Emphasis was placed on the Latino population.

**Results:**

Based on parent-reported sleep measures, 83.3% of children met sleep guidelines (defined as 9–12 h per night), compared to 14.7% based on accelerometry (*ρ* = −0.036, *p* = 0.711). Average sleep duration significantly differed between parent reports (9.58 h, SD = 1.42) and actigraphy (8.32 h, SD = 0.70; *ρ* = 0.405, *p* < 0.001). There were no discrepancies between objective and subjective reported sleep latency and WASO, although paired tests indicated significant within-person differences in WASO (*p* < .001). Finally, there were significant discrepancies in sleep duration between Latinos and non-Latinos, with Latino caregivers reporting significantly shorter sleep and their children experiencing shorter sleep duration as measured via actigraphy, and being less likely to meet sleep guidelines.

**Conclusion:**

These findings suggest that there is a discrepancy between perceived and objectively measured sleep. It also suggests discrepancies in sleep duration between Latino and non-Latino children, and differences in parental knowledge of sleep behaviors between Latinos and non-Latinos.

## Introduction

Sleep is an essential component of children's health and development (1), contributing to their brain development (2, 3), cognitive and psychiatric health (4, 5), and learning ability (6). Additionally, adequate sleep is associated with reduced cardiometabolic risk in children and adolescents (7). Nevertheless, inadequate sleep in children and youth is a growing public health concern (8), as estimates of sleep duration show a downward trend across time (9). A systematic review of the literature by Matricciani et al. found that between 1905 and 2008, there was a decrease of approximately one hour of sleep per night in children aged 5–18 years. Furthermore, a report by the Centers for Disease Control and Prevention (CDC) estimated that one-third of children aged four months to 17 years sleep less than recommended for their age (10). Short sleep duration in the pediatric population is particularly concerning due to an increased risk of obesity (11, 12), attention-deficit/hyperactivity disorder (ADHD) (13), and mental health problems (14).

Not only is the duration of sleep important for children's health (15), but other measures of sleep quality, such as sleep latency (i.e., number of minutes it takes to fall asleep after lying in bed) and wake after sleep onset (WASO, i.e., the number of minutes one is awake during the night) have also been widely used in children's sleep research (16–19). Short sleep latency is associated with excessive daytime sleepiness and narcolepsy, while long sleep latency is associated with insomnia (20, 21). High levels of WASO may be indicative of fragmented sleep (22, 23), which could impact sleep duration, and in turn, affect children's development (24, 25).

In Rhode Island, 69.2% of youth aged 4–17 years met sleep guidelines from 2022 to 2023, and between 22.8 and 34.6% of children aged 6–11 years slept an inadequate amount for their age group (26, 27). The percentage of children aged 4–14 years with insufficient sleep in Rhode Island (33.4%) is comparable to that of the United States (35%) (28). While survey data reveal a high prevalence of inadequate sleep duration for children in Rhode Island, data to evaluate children's sleep quality, such as sleep latency and WASO, are lacking. The present study aimed to expand on the existing knowledge of sleep quality and quantity in this age group and geographic region.

Considering the importance of sleep in children, parents must have an accurate awareness of their child's sleep habits. Nevertheless, research suggests parents generally have poor knowledge of their child's sleep (29). A study by Dayyat et al. (30) assessing parent reports and actigraphy in children who were both healthy and displaying sleep-related symptomatology found parents in all groups overestimated sleep duration by over one hour. Similarly, Mazza, Bastuji, and Rey (31) found that when having parents and children self-report the child's own sleep outcomes, parents and children both overestimated sleep duration by approximately 36 min and 92 min, respectively, when compared to actigraphy. While parents have more accurate knowledge of bedtimes and wake times, there are greater discrepancies in parental knowledge of total sleep time and time awake in bed (sleep latency and WASO) (32). Similar findings on the discrepancy between subjective and objective measures of sleep have been reported in adolescents as well (33). In contrast with the aforementioned studies, the current analysis seeks to further understand parental perspectives of sleep by asking them to report whether they believe their child is sleeping the correct amount of time. As parental perceptions of adequate sleep may impact sleep practices, parental perspectives are of particular relevance.

The current study places emphasis on the Latino population. To date, the majority of sleep research has been conducted on non-Hispanic White individuals (34). Studying sleep in Latino children is especially important as they are disproportionately affected by cultural and socioeconomic disparities that affect health outcomes and access to health care (35, 36). A review by Guglielmo et al. (37) found racial/ethnic disparities in sleep outcomes between children and adolescents, where White children generally had better and longer sleep than their Hispanic and Black counterparts. Minority children have shorter sleep duration and later bedtimes than their White counterparts (38). Additionally, studies show that between one-third and 40% of Hispanic/Latino children do not meet sleep duration guidelines (10, 39). Moreover, Hispanic youth have been found to have a later chronotype and greater social jet lag in comparison to non-Hispanic youth (40). Studies using objective measures of sleep in Latino children are limited and have been encouraged by previous reviews (41). This study aims to expand the existing knowledge of the Latino pediatric population using objective measures of sleep.

This study aimed to further investigate the difference between parent-reported and actigraphy-derived sleep outcomes in early elementary-aged children. We hypothesized a significant discrepancy between parent-reported (subjective) and actigraphy-derived (objective) sleep outcomes, where parents would over-report sleep duration and under-report sleep latency (i.e., number of minutes it took the participant to fall asleep) and wake after sleep onset (WASO, i.e., number of minutes the participant was awake during the night). Furthermore, we hypothesized discrepancies between Latino and non-Latino children in outcomes of both sleep quality and quantity, where Latino children would experience shorter sleep duration and poorer sleep quality outcomes. Given previous studies’ findings that parents underestimate children's time awake in bed, we hypothesized that parent-reported sleep duration would be more associated with time in bed than total sleep time. Additionally, the study aimed to expand on the existing knowledge of sleep quality and quantity in children in grades 1, 2, and 3 in Rhode Island and also expand the existing knowledge of the Latino pediatric population using objective measures of sleep.

## Methods

### Study setting

The study was conducted in the state of Rhode Island, a northeastern US state with one million residents (42). Rhode Island is characterized by inadequate sleep in children and adolescents, with only 69% of youth ages 4–17 meeting sleep guidelines (27), providing an appropriate setting for this study. Additionally, Rhode Island is characterized by racial and ethnic diversity, with approximately 25% of the population exclusively identifying as either Hispanic/Latino, Black/African American, or Asian. The study was conducted between the fall of 2021 and the spring of 2024.

### Participants

Participants are part of the Project Greenspace, Sleep, and Mental Health (G-SPACE) study (43). The primary aim of Project G-SPACE is to investigate the influence of exposure to green spaces on the health of elementary school children. The project seeks to recruit children in grades one, two, and three in the state of Rhode Island. Additional inclusion criteria include: 1) legal parent or guardian or recognized caregiver (e.g., grandparent) 18 years of age or older and 2) English or Spanish-speaking. Exclusion criteria included 1) disability that limits physical activity, 2) seizures or other neurological or neuromuscular disorders, and 3) medical conditions that limit participation in the study. Participants with neurodevelopmental disorders were not excluded. Additionally, all participants in the current sample attended traditional, in-person schooling. The current analysis is composed of those who participated from the fall of 2021 to the spring of 2024 (*n* = 128). Data collection procedures for project G-SPACE are ongoing.

### Procedures

Participants were recruited through several methods. Flyers were distributed to local elementary schools or community organizations for children and caregivers to receive, as well as at community events. Caregivers had the option to return the flyers to their child's school or organization with their information to be contacted by the research team or use the research team's information to directly contact them. Additionally, radio advertisements on local stations were also used to recruit participants. In this case, parents received the team's phone number and email address in the advertisement and directly contacted the team. Finally, advertisements on social media platforms such as Facebook and Instagram were also used to recruit participants. All recruitment materials were translated into Spanish for the accessibility of Spanish-speaking families. After contact, participants are screened for eligibility. Upon determining eligibility, two in-person visits are conducted to obtain consent and orient families to study procedures. After visit one, participants wear an accelerometer for seven days, during which parents complete morning and nightly surveys. After the seven-day period, visit two takes place to retrieve the devices from the participants. Data collection occurred during the academic year, during non-school-vacation periods, and during daylight saving time. See Grigsby-Toussaint et al. (43) for more information on procedures and eligibility.

This study has been approved by the Brown Institutional Review Board, protocol #2105002996, which includes procedures for parental consent and child assent.

### Measures

#### Objective sleep

Objective measures of sleep were obtained by participants wearing a multimodal Actiwatch Spectrum Plus (Philips Respironics, Inc.; Murrysville, PA, USA) on the non-dominant wrist, which recorded activity in 15 s epochs at a medium sensitivity threshold. Parents were instructed to have their child wear the actiwatch at all times except during the possibility of the device getting wet. The following measures were obtained: total time in bed (TIB; time between bedtime and waketime (17), sleep duration (total sleep time; TST), sleep latency (i.e., number of minutes it took the participant to fall asleep), and wake after sleep onset (WASO, i.e., number of minutes the participant was awake during the night). Objective total sleep time will be referencing sleep duration in the current analysis. Additionally, bedtime and wake time were also obtained. Sleep parameters were averaged across nights, and weekday and weekend data were pooled. All measures were obtained from Philips’ clinicians report, which provided automatically scored sleep parameters via the Philips Actiware software version 6.3. Additional analyses were completed at the daily level. These analyses were completed using manually scored actigraphy data and were uncoupled by weekday and weekend.

#### Subjective sleep

Subjective measures of sleep were obtained by parents completing the Children's Sleep Habits Questionnaire (CSHQ) (44) at baseline. Firstly, parents were asked to provide their “Child's usual amount of sleep each night (no naps) in hours and minutes (i.e., sleep duration).” This was modified from the original question of “Child's usual amount of sleep each day (combining nighttime sleep and naps).” This was the only modification to the CSHQ. Secondly, they reported whether their child, on average, falls asleep in under 20 min (i.e., sleep latency), which they reported using the following options: “Usually (5–7 days)”, “Sometimes (2–4 days),” and “Rarely (0–1 days).” They also reported with a “yes” or “no” whether they considered this a sleep habit problem. Thirdly, parents reported the number of minutes that a night waking generally lasts (i.e., WASO). Additionally, they were asked whether they believed their child generally “sleeps the right amount,” which they reported using the following options: “Usually (5–7 days),” “Sometimes (2–4 days),” and “Rarely (0–1 days).” They also reported with a “yes” or “no” whether this was a sleep habit problem. In addition to completing the CSHQ at baseline, parents completed sleep diaries in which they reported perceived sleep duration daily through the following question: “How much sleep do you think your child got last night? Answer in hours and minutes.” Daily-level associations between subjective and objective sleep measures were examined using this daily question.

Both subjective and objective sleep duration measures were compared to the national child sleep guidelines for children aged 6–12 years of 9–12 h established by the American Academy of Sleep Medicine (AASM) and endorsed by the American Academy of Pediatrics (45) and the National Sleep Foundation (52).

### Statistical methods

All statistical analyses, including the descriptive statistics, comparison statistics, and correlation statistics, were performed using the software R (53). All statistical analyses were conducted on the aggregate sample and stratified by ethnicity (Latino and non-Latino). The descriptive statistics include mean and standard deviation for continuous variables, and count and frequencies for categorical data. The significance tests include a Student's *t*-test (two-sample) and Chi-squared or Fisher's test for categorical variables (depending on sample size in each cell) and non-parametrics as appropriate. Fisher's test was used when cell sizes were <5. Parametric tests were used only when the normality of the outcome variable was reasonable (skewness, kurtosis, and graphical methods were used to establish normality).

Parents were asked whether their child falls asleep in under 20 min and if they sleep the right amount.

These two variables were coded using three levels: usually (5–7 days), sometimes (2–4 days), and rarely (0–1) days. Variables were re-coded to binary, where “usually” indicated that the child fell asleep in under 20 min and slept the right amount.

A Spearman's rank correlation was conducted to understand if there is a correlation between the subjectively measured (parent-reported) and objectively measured (actigraphy monitor) sleep outcome data within the sample. The rho coefficient (ρ) and *p*-value are reported in Tables 2, 3. Furthermore, between-sample comparison was performed using Student's *t*-test (two-sample) and chi-square tests (*χ*^2^) or Fisher's test (depending on sample size in each cell). To understand within-subject agreement between objective and subjective measures of sleep, paired tests were conducted (parametric or non-parametric as appropriate).The significance threshold used across all analyses was an alpha of 0.05.

Daily-level associations between parent-reported (sleep diary) data and objectively measured (actigraphy) data were examined using non-parametric correlations and paired tests. The goal was to examine whether the pattern of findings was similar between self-reported and device-measured sleep. Data was considered at the daily level (disaggregated by weekday vs. weekend).

## Results

Of the full sample of 128 participants, 24 were excluded due to not completing study procedures (*n* = 10), having insufficient device (*n* = 9) or survey data (*n* = 5), and being identified as statistical outliers (*n* = 2). Participants were categorized as not completing study procedures if they dropped out of the study. Participants were also categorized as having insufficient device data if they wore the actiwatch for one night or less and insufficient survey data if not all survey questions were completed. The final analytic sample consisted of *n* = 102 children, with a mean age of 7.63 years (SD = 0.99, range 6–10 years), and more than half of the sample was female (57.8%). A majority of the participants had an annual household income of $150,000 or more (28.4%), followed by 18.6% between $100,000 and $149,999, and 13.7% in the lowest income category of less than $5,000. The sample was primarily White (46.1%) and non-Hispanic/Latino (55.9%). Further demographic characteristics of the sample can be seen in Table 1.

**Table 1:** Sociodemographic characteristics of project G-SPACE analytic sample.

	Study sample *N* = 102	Latino ethnicity *n* = 45	Non-Latino ethnicity *n* = 57	*p*-value (Latino vs. non-Latino)
Age [Mean, (SD)]				0.962
	7.63 (0.99)	7.62 (0.89)	7.63 (1.08)	
Age (*N*, %)				0.408
6	14 (13.7%)	4 (8.9%)	10 (17.5%)	
7	33 (32.4%)	17 (37.8%)	16 (28.1%)	
8	33 (32.4%)	16 (35.6%)	17 (29.8%)	
9+	22 (21.6%)	8 (17.8%)	14 (24.6%)	
Gender (*N*, %)				0.951
Female	59 (57.8%)	26 (57.8%)	33 (57.9%)	
Male	41 (40.2%)	19 (42.2%)	22 (38.6%)	
Non-binary	1 (1.0%)	0 (0%)	1 (1.8%)	
Transgender Female	1 (1.0%)	0 (0%)	1 (1.8%)	
Annual Household Income, $ (*N*, %)				0.0005
Less than 5,000	14 (13.7%)	12 (26.7%)	2 (3.5%)	
5,000–9,999	2 (2.0%)	1 (2.2%)	1 (1.8%)	
10,000–14,999	2 (2.0%)	1 (2.2%)	1 (1.8%)	
20,000–25,999	11 (10.8%)	9 (20.0%)	2 (3.5%)	
26,000–49,999	11 (10.8%)	8 (17.8%)	3 (5.2%)	
50,000–74,999	8 (7.8%)	4 (8.9%)	4 (7.0%)	
75,000–99,999	6 (5.9%)	1 (2.2%)	5 (8.8%)	
100,000–149,999	19 (18.6%)	9 (20.0%)	10 (17.5%)	
150,000 or more	29 (28.4%)	0 (0%)	29 (50.9%)	
Race/Ethnicity (*N*, %)				*<*2.2*10^−16^
White	47 (46.1%)	2 (4.3%)	45 (78.9%)	
Asian	4 (3.9%)	2 (4.4%)	2 (3.5%)	
Black/African American	5 (4.9%)	2 (4.4%)	3 (5.3%)	
American Indian or Alaskan Native	1 (1.0%)	1 (2.2%)	0 (0%)	
More than one	7 (6.9%)	0 (0%)	7 (12.3%)	
Other	38 (37.3%)	38 (84.4%)	0 (0%)	
Ethnicity (*N*, %)				
Hispanic/Latino	45 (44.1%)	45 (100%)	0 (0%)	

*Race as determined by (“Which categories describe your child? Select all that apply”).

*Latino ethnicity as determined by (“Is your child Hispanic/Latino?” Yes or No).

### Objective vs. subjective sleep outcomes by ethnicity

#### Total sleep time

In the overall sample, the average parent-reported sleep duration was 9.58 h (SD = 1.42 h), while the average objective TST was 8.32 h (SD = 0.70 h). There was a statistically significant positive correlation between the objective and subjective measures in the overall sample; as one variable increases the other does as well (Table 2; *ρ* = 0.405, *p* < .001). Furthermore, there was a significant difference in parent-reported sleep duration between the Latino and non-Latino subgroups, with Latino parents reporting an average of 9.08 h per night (SD = 1.88 h) and non-Latino parents reporting an average of 9.98 h per night (SD = 0.72 h; t = −3.019, *p* = .004). Similarly, there was a significant difference between objective TST in the Latino subgroup (8.04 h, SD = 0.61 h) and the non-Latino subgroup (8.53 h, SD = 0.69 h; t = −3.790, *p* < 0.001). Lastly, there was significant within-group correlation for parent-reported vs. objective TST for the non-Latino sample (*ρ* = 0.409, *p* = .002). There was some correlation between the objective and subjective measures within the Latino sample, but the correlation was not statistically significant (*ρ* = 0.282, *p* = .060). Lastly, there was a statistically significant difference between the parent-reported and objectively measured sleep durations within the aggregate, Latino, and non-Latino samples.

When examining daily level associations using parent-reported daily sleep diaries and uncoupled weekday from weekend data, significant correlations between daily diary-reported sleep and actigraphy-measured sleep across all days were present where correlation coefficients ranged between.29 and.49 with all *p* values <.01. Thus indicating small-to-moderate associations between these two measures of sleep duration. Within-person differences between daily self-report and device-measured sleep were calculated and showed significant differences within-person (*p* < .01) such that self-report was significantly higher on average compared to device-measured (9 h, 43 min vs. 8 h, 59 min, respectively).

#### Time in bed

When comparing the parent-reported sleep duration and the objectively measured time in bed, there was a statistically significant correlation for the overall sample and the non-Latino subsample (Table 2; Total sample: *ρ* = 0.409, *p* < .001; non-Latino sample: *ρ* = 0.509, *p* < .001). There was no statistically significant difference between the objective measure and the parent-reported measure for the overall sample and the Latino sample (Table 2; Total sample: t = 0.694, *p* = 0.489; Latino sample: t = −0.343, *p* = 0.732). Furthermore, there was a significant difference between objective total time in bed in the Latino subgroup (9.19 h, SD = 0.76 h) and the non-Latino subgroup (9.69 h, SD = 0.80 h; t = −3.269, *p* = 0.002).

**Table 2:** Descriptive statistics of objective vs. subjective sleep measures, stratified by Latino/non-Latino ethnicity^a^.

	Overall population *N* = 102	Latino *n* = 45	Non-Latino *n* = 57	Comparison Latino vs. non-Latino
Total sleep time, hours	Mean (SD)			
Parent reported total sleep time	9.58 (1.42)	9.08 (1.88)	9.98 (0.72)	t = −3.019, ***p*** **=** **.004**
Objective total sleep time	8.32 (0.70)	8.04 (0.61)	8.53 (0.69)	t = −3.790, ***p*** **<** **.001**
Correlation: parent-reported vs. objective	*ρ* = 0.405, ***p*** **<** **.001**	*ρ* = 0.282, *p* = .060	*ρ* = 0.409, ***p*** **=** **.002**	
Comparison (*t*-test)	t = 8.075, ***p*** **<** **.001**	t = 3.529, ***p*** **<** **.001**	t = 10.977, ***p*** **<** **.001**	
Time in bed, hours	Mean (SD)			
Parent-reported total sleep time	9.58 (1.42)	9.08 (1.88)	9.98 (0.72)	t = −3.019, ***p*** **=** **.004**
Objective time in bed	9.47 (0.82)	9.19 (0.76)	9.69 (0.80)	t = −3.269, ***p*** **=** **.002**
Correlation: parent-reported vs. objective	*ρ* = 0.409, ***p*** **<** **.001**	*ρ* = 0.177, *p* = .245	*ρ* = 0.509, ***p*** **<** **.001**	
Comparison (*t*-test)	t = 0.694, *p* = 0.489	t = −0.343, *p* = 0.732	t = 1.999, ***p*** **=** **0.048**	
WASO (minutes)	Mean (SD)			
Parent-reported WASO	4.78 (11.28)	3.16 (9.06)	6.06 (12.70)	t = −1.347, *p* = .181
Objective WASO	38.27 (14.05)	37.78 (16.74)	38.65 (11.64)	t = −0.296, *p* = .768
Correlation: parent-reported vs. objective	*ρ* = 0.035, *p* = .724	*ρ* = −0.124, *p* = .416	*ρ* = 0.124, *p* = .358	
Comparison (*t*-test)	t = −18.772, ***p*** **<** **.001**	t = −12.205, ***p*** **<** **.001**	t = −14.283, ***p*** **<** **.001**	
Sleep latency: 20 min or less	*N* (%)			
Parent-reported sleep latency	71 (69.6%)	34 (75.6%)	37 (64.9%)	*χ*^2^ = 0.127, *p* = 0.722
Objective sleep latency	85 (83.3%)	37 (82.2%)	48 (84.2%)	*χ*^2^ = 1.424, *p* = .233
Correlation: parent-reported vs. objective	*ρ* = 0.162, *p* = 0.104	*ρ* = 0.277, *p* = 0.066	*ρ* = 0.085, *p* = 0.530	
Comparison (chi-square)	*χ*^2^ = 1.256, *p* = 0.262	*χ*^2^ = 0.127, *p* = 0.722	*χ*^2^ = 1.424, *p* = 0.233	

Bold values indicate significant results (*p* < .05).

^a^ Latino ethnicity as determined by (“Is your child Hispanic/Latino?” Yes or No).

#### Wake after sleep onset

The average parent reported WASO across both ethnicity subgroups was 4.78 min (SD = 11.28 min), and the average objective WASO was 38.27 min (SD = 14.05 min). There were no statistically significant differences in parent-reported WASO across the Latino (3.16 min, SD = 9.06 min) and non-Latino subgroups (6.06, SD = 12.70 min; t = −1.3474, *p* = 0.181), or in objective WASO across the Latino (37.78 min, SD = 16.74 min) and non-Latino subgroups (38.65 min, SD = 11.64 min; t = −0.296, *p* = 0.768). Furthermore, no within-group correlation was found between parent-reported WASO and objectively measured WASO for either the Latino or non-Latino samples (Latino: *ρ* = −0.124, *p* = 0.416; non-Latino: *ρ* = 0.124, *p* = 0.358). However, there was a statistically significant difference between the two measures for aggregate, Latino, and non-Latino samples.

#### Sleep latency

The majority of the sample had a parent-reported sleep latency of 20 min or less

(69.6%), while 83.3% of all children had an objective sleep latency of 20 min or less (*ρ* = 0.162, *p* = .104). There was no statistically significant difference detected between the Latino and non-Latino sample for either the parent-reported sleep latency (*χ*^2^ = 0.127, *p* = .722) and the objectively measured sleep latency (*χ*^2^ = 1.424, *p* = .233). Furthermore, there were no significant within-group estimates when looking at the correlation between the objective measure of sleep latency and the parent-reported sleep latency (Latino: *ρ* = 0.277, *p* = .066; non-Latino: *ρ* = 0.085, *p* = .530). Similarly, there was no significant difference between the two reported measures (Latino: *χ*^2^ = 0.127, *p* = 0.722; non-Latino: *χ*^2^ = 1.424, *p* = 0.233).

### Meeting sleep guidelines

#### Parent-reported vs. total sleep time

The percentage of parents that reported they believed their child slept the right amount was 83.3%, while only 14.7% of children met sleep guidelines of 9–12 h of sleep when measured via actigraphy (Table 3; *ρ* = −0.037, *p* = .711). No significant differences were seen in parent-reported sleep guidelines between the Latino and non-Latino subgroups, with 88.9% of Latino parents reporting that their child slept the right amount, compared to 78.9% of non-Latino parents (*χ*^2^ = 0.294, *p* = .588). There was a significant difference in objectively meeting sleep guidelines between the Latino and non-Latino subgroups: 4.4% of Latino children met sleep guidelines according to Total Sleep Time, compared to 22.8% of non-Latino children (*χ*^2^ = 8.067, *p* = .005). Furthermore, there were no significant within-group correlations between parents reporting that their child met guidelines and the number of children who met national guidelines objectively (Latino: *ρ* = 0.076, *p* = .619; non-Latino: *ρ* = −0.027, *p* = .842). However, there is a statistically significant within-person difference between the two reported measures among both Latinos and non-Latinos (Latino: *χ*2 = 34.38, *p* < 0.001; non-Latino: *χ*^2^ = 17.66, *p* < 0.001), indicating an overestimation from parents.

**Table 3:** Meeting AASM national sleep guidelines of 9–12 of sleep per night, with parental report of whether child “sleeps the right amount” per night and objective total sleep time, stratified by Latino/non-Latino ethnicity^a^.

	Overall population *N* = 102	Latino *n* = 45	Non-Latino *n* = 57	Comparison Latino vs. non-Latino
Meets AASM guidelines- total sleep time				
Parent-reported	85 (83.3%)	40 (88.9%)	45 (78.9%)	*χ*^2^ = 0.294, *p* = 0.588
Objective	15 (14.7%)	2 (4.4%)	13 (22.8%)	*χ*^2^ = 8.067, ***p*** **=** **0.0045**
Correlation: parent-reported vs. objective	*ρ* = −0.037, *p* = 0.711	*ρ* = 0.076, *p* = 0.619	*ρ* = −0.027, *p* = 0.842	
Comparison (chi-square)	*χ*^2^ = 49, ***p*** **<** **.001**	*χ*^2^ = 34.38, ***p*** **<** **.001**	*χ*^2^ = 17.66, ***p*** **<** **.001**	
Only among those with parents who believed their child “slept the right amount”	*n* *=* *85*	*n* *=* *40*	*n* *=* *45*	
Objective measure	12 (14.1%)	2 (5.0%)	10 (22.2%)	*χ*^2^ = 5.333, ***p*** **=** **0.021**
Average TST	8.34 (0.69%)	8.07 (0.64%)	8.59 (0.64%)	t = −3.732, ***p*** **<** **.001**
Average TIB	9.50 (0.81%)	9.19 (0.75%)	9.78 (0.76%)	t = −3.603, ***p*** **<** **.001**

Bold values indicate significant results (*p* < .05).

^a^ Latino ethnicity as determined by (“Is your child Hispanic/Latino?” Yes or No).

#### Parent-reported sleep duration problem vs. objectively measured sleep duration

The percentage of parents that reported a sleep habit problem for sleep duration was 46.1% (i.e., did not “sleep the right amount”), compared to 85.3% of children that did not meet the sleep guidelines of 9–12 h measured through actigraphy. Significant differences were seen in parent-reported sleep habit problems in the Latino and non-Latino subgroups, with 68.9% of Latino parents reporting that their child had a sleep duration problem, compared to 28.1% of non-Latino parents (Table 4; *χ*^2^ = 4.787, *p* = 0.029). There was no significant difference in objectively failing to meet sleep guidelines between the Latino and non-Latino subgroups. Furthermore, there were no significant within-group correlations between parent-reported sleep duration habits and the number of children who did not meet national guidelines objectively. Lastly, there is a significant within-person effect between parent-reported problematic sleep and objectively measured sleep duration for the aggregate sample and non-Latino sample (full sample: *χ*^2^ = 11.94, *p* < 0.001; non- Latino sample: *χ*^2^ = 13.067, *p* < 0.001).

**Table 4:** Meeting AASM national sleep guidelines of 9–12 of sleep per night, with parental report of whether child “sleeps the right amount” per night is a sleep habit problem and objective total sleep time, stratified by latino/non-latino ethnicity^a^.

	Overall population *N* = 102	Latino *n* = 45	Non-Latino *n* = 57	Comparison Latino vs. non-Latino
Meets AASM guidelines: parent reported sleep habit problem vs. total sleep time				
Parent-reported	47 (46.1%)	31 (68.9%)	16 (28.1%)	*χ*^2^ = 4.787, ***p*** **=** **0.029**
Objective	87 (85.3%)	43 (95.6%)	44 (77.2%)	*χ*^2^ = 0.011, *p* = 0.915
Correlation: parent-reported vs. objective	*ρ* = −0.162, *p* = 0.104	*ρ* = 0.145, *p* = 0.342	*ρ* = −0.153, *p* = 0.254	
Comparison (chi-square)	*χ*^2^ = 11.94, ***p*** **<** **0.001**	*χ*^2^ = 1.946, *p* = 0.163	*χ*^2^ = 13.067, ***p*** **<** **0.001**	

Bold values indicate significant results (*p* < .05).

^a^Latino ethnicity as determined by (“Is your child Hispanic/Latino?” Yes or No)".

#### Parent-reported sleep latency problem vs. objectively measured sleep latency

Approximately half of the sample (49.0%) had a parent report that the child had a sleep habit problem for sleep latency (i.e., not falling asleep in under 20 min), while 16.7% of all children had an objective sleep latency of more than 20 min. There was no statistically significant difference detected between the Latino and non-Latino sample for either the parent-reported problematic sleep latency habit (Table 5; *χ*^2^ = 1.28, *p* = 0.258) or the objectively measured sleep latency (*χ*^2^ = 0.059, *p* = 0.808). Furthermore, there were no significant within-group estimates for the correlation between objectively measured sleep latency and the parent-reported sleep latency problem (Latino: *ρ* = 0.019, *p* = 0.902; non-Latino: *ρ* = −0.168, *p* = 0.212). Lastly, within-person associations between parent-reported problematic sleep latency and the objective sleep latency measure suggested significant effects (full sample: *χ*^2^ = 16.254, *p* < 0.001; Latino sample: *χ*^2^ = 11.92, *p* < 0.001; non-Latino sample: *χ*^2^ = 4.8, *p* = 0.0285).

**Table 5:** Meeting AASM national sleep guidelines of 9–12 of sleep per night, with parental report of whether child “falls asleep in under 20 min” is a sleep habit problem and objective total sleep time, stratified by latino/non-latino ethnicity^a^.

	Overall population *N* = 102	Latino *n* = 45	Non-Latino *n* = 57	Comparison Latino vs. non-Latino
Sleep latency, 20 min or less: parent-reported sleep habit problem vs. objective				
Parent-reported	50 (49.0%)	29 (64.4%)	21 (36.8%)	*χ*^2^ = 1.28, *p* = 0.258
Objective	17 (16.7%)	8 (17.8%)	9 (15.8%)	*χ*^2^ = 0.059, *p* = 0.808
Correlation: parent-reported vs. objective	*ρ* = −0.088, *p* = 0.381	*ρ* = 0.019, *p* = 0.902	*ρ* = −0.168, *p* = 0.212	
Comparison (chi-square)	*χ*^2^ = 16.254, ***p*** **<** **0.001**	*χ*^2^ = 11.92, ***p*** **<** **0.001**	*χ*^2^ = 4.8, ***p*** **=** **0.0285**	

Bold (*p*) values indicate statistical significance.

^a^Latino ethnicity as determined by (“Is your child Hispanic/Latino?” Yes or No)".

## Discussion

This study indicates that there is a discrepancy between actigraphy-derived and parent-reported sleep duration and WASO, but not sleep latency. In this sample, while 83.3% of children met the American Academy of Pediatrics-endorsed sleep guidelines for their age group through parent reports, only 14.7% of children met the guidelines according to actigraphy, with significant disparities in meeting sleep guidelines by ethnicity. Using actigraphy-derived metrics, non-Latino children were significantly more likely to meet sleep guidelines (22.8%) than Latino children (4.4%). There were discrepancies in actigraphy-derived sleep duration by ethnicity, where Latino children experienced significantly shorter sleep than non-Latinos. Interestingly, when comparing parental reports of sleep duration to total time in bed via the accelerometer, parental reports for the non-Latino subgroup were still significantly higher than objectively obtained time in bed. It is important to note, however, that TST and TIB are distinct constructs, where TIB encompasses the time between bedtime and waketime and thus may not be a comparable measure to parental-reported sleep duration. However, it does provide broader context that aids in understanding how parental perspectives of sleep compare to objective sleep outcomes. As previously mentioned, parents generally have accurate knowledge of bedtimes and wake times but lack knowledge of time spent awake after bedtime (32). The current analysis also found discrepancies between parent-reported and objective WASO. There were significant differences between objective and subjective WASO that remained present in both Latino and non-Latino participants after stratification, though there were no between-group differences in parent-reported and objectively obtained WASO. When comparing parental-reported sleep duration problems and objectively not meeting sleep guidelines, a significant effect was present for the aggregate sample. This effect was present only in the non-Latino subgroup after stratification. This may suggest that the non-Latino sample is underestimating problems with sleep duration habits. Additionally, while there were no significant differences present between objective and subjective sleep latency, when assessing parent-reported sleep latency problems (i.e., reported problem with falling asleep in under 20 min) and objectively not falling asleep in under 20 min, within-person associations were present in the aggregate sample and in both subgroups. This suggests that while parents have accurate awareness of sleep latency, they may be overreporting sleep latency problems. Importantly, the discrepancy between subjective and objective sleep duration was still present when using daily-level parent reports. Overall, these findings support the current literature's findings on the discrepancy in sleep outcomes between Latino and non-Latino children. Additionally, they suggest that overall discrepancies between objectively measured sleep and parental perceptions of a child's sleep exist, and they may be a function of time spent awake in bed (i.e., WASO).

### Latino vs. non-Latino differences

Significant differences emerged between Latino and non-Latino participants. There were significant differences in sleep duration between Latino and non-Latino participants, where Latino caregivers reported significantly shorter sleep duration and their children experienced significantly shorter sleep via actigraphy. Furthermore, only 4.4% of Latino participants met sleep guidelines according to actigraphy (22.8% for non-Latinos); this is a lower percentage than other studies have found using parent-reported data (39). When comparing WASO between Latino and non-Latino participants, there were no between-group differences present; however, there were significant differences between subjective and objective sleep outcomes in both groups. No differences emerged when comparing sleep latency between and within both groups. Furthermore, when assessing meeting sleep guidelines, there were no significant differences in the number of parents reporting whether their child “sleeps the right amount,” however, non-Latino children were significantly more likely to meet guidelines via actigraphy using TST. However, discrepancies between subjective and objective reports were found in both groups. Interestingly, while there were no differences between groups in caregivers reporting whether their child “sleeps the right amount,” when reporting whether sleeping the right amount was a sleep habit problem, Latino caregivers were significantly more likely to report “yes.” However, there were significant differences between reporting “yes” and objectively not meeting sleep guidelines in the non-Latino subgroup, indicating that non-Latino caregivers may be under-reporting sleep duration problems. As discussed, both Latino and non-Latino caregivers overreported sleep latency problems. Moreover, analyses on meeting sleep guidelines among parents who believe their child “sleeps the right amount” showed discrepancies, where non-Latino participants were still significantly more likely to meet sleep guidelines and had significantly longer TST and TIB via actigraphy. These findings suggest that while Latino children experience poor sleep duration and are less likely to meet sleep guidelines, both Latino and non-Latino caregivers have comparable awareness of their child's time spent awake after bedtime. However, there are some differences in parental perspectives of sleep duration, where Latino parents report similarly as to whether their child sleeps the right amount while being less likely to meet sleep guidelines, while non-Latino caregivers also under-report sleep duration problems. Additionally, there were no significant differences in parental-reported sleep duration and objectively obtained TIB in the Latino subgroup. This suggests that Latino caregivers may be considering TIB when thinking about their child's sleep duration, which could influence awareness of sleep.

There are cultural factors that may explain the differences in sleep behaviors and outcomes between Latino and non-Latino participants. For example, one study found that Latino children in this age group generally have later bedtimes than non-Latino children (46). Latino families are also more likely to practice co-sleeping or bed-sharing than non-Latinos (47), which could impact parental awareness of sleep. A study investigating the influence of the physical home environment on sleep in children found that room-sharing with an adult was the most important predictor of sleep timing, sleep duration, and parent-reported sleep problems (48). Children in the study who shared a room with an adult had later bedtimes, shorter sleep duration, and more parent-reported problems. Latino families are also more likely to live in multi-generational homes (49), which could also potentially alter sleep outcomes. For example, Liu, Liu, and Wang (50) investigated bed sharing and sleep habits among school-aged children and found an increased likelihood of bed sharing in crowded households, which was associated with sleep anxiety and daytime sleepiness. However, it is important to note that these factors were not measured in the present analysis and thus are not inferred as causal. In the past, many quantitative studies investigating sleep in Latino children have relied on parent reports (41). Future research should investigate whether there are cultural implications of addressing sleep in children via parent reports. Future research should also investigate mediators that may account for the discrepancy in sleep outcomes between Latino and non-Latino children.

### Strengths and limitations

This study is one of the few to compare objectively derived sleep outcomes in children with subjective parent reports among Latino and non-Latino elementary school children. The data collection period of one week allowed for the capture of variability across different days. Additionally, stratification by race/ethnicity allowed for disparities in sleep outcomes among Latinos and non-Latinos to be recognized and addressed. Finally, additional analyses for sleep duration were completed using temporally aligned parental reports and manually scored sleep data uncoupled by weekday and weekend. However, there are several limitations to the study. This analysis did not take under consideration pre-existing health conditions or mental health status for participants or caregivers. Different types of caregivers were also not distinguished within this analysis (i.e., parents vs. non-parent caregivers). Furthermore, we did not account for other social or environmental factors that could also have an impact on sleep (e.g., parenting practices or neighborhood characteristics, respectively). We also did not conduct stratified analyses by season. Moreover, actigraphy-derived sleep latency and wake after sleep onset are less accurate given the difficulty of distinguishing between sleep and quiet restfulness using actigraphy (51,52).

## Conclusions

When comparing parent-reported and actigraphy-obtained sleep outcomes, we found that parents generally overestimate their child's sleep duration and underestimate wake after sleep onset (WASO). Additionally, while they have accurate awareness of sleep latency, they overreport sleep latency problems. When stratifying by ethnicity, these discrepancies remained present in both groups. There were discrepancies in sleep duration between Latinos and non-Latinos, where Latinos experienced shorter sleep. We also found that only 14.7% of children met the American Academy of Pediatrics-endorsed guidelines for their age group when measured via actigraphy, with significant discrepancies between Latino and non-Latino participants. These discrepancies between parent-reported and objective sleep measures present challenges to consider in future observational studies of sleep quality. Future research should also further investigate the mechanisms by which the discrepancy between parental perceptions of child sleep and objectively measured sleep outcomes exists.

## Data Availability

The datasets presented in this article are not readily available because the datasets generated and/or analyzed during the current study are not publicly available as we are continuing to actively recruit participants but are available from the corresponding author on reasonable request. Requests to access the datasets should be directed to diana_grigsby-toussaint@brown.edu.

## References

[1] Matricciani L Paquet C Galland B Short M Olds T. Children’s sleep and health: a meta-review. Sleep Med Rev. (2019) 46:136–50. 10.1016/j.smrv.2019.04.011 31121414

[2] Jiang F. Sleep and early brain development. Ann Nutr Metab. (2019) 75(Suppl. 1):44–54. 10.1159/000508055 32564032

[3] Lokhandwala S Spencer RM. Relations between sleep patterns early in life and brain development: a review. Dev Cogn Neurosci. (2022) 56:101130. 10.1016/j.dcn.2022.101130 35779333 PMC9254005

[4] Cheng W Rolls E Gong W Du J Zhang J Zhang XY Sleep duration, brain structure, and psychiatric and cognitive problems in children. Mol Psychiatry. (2021) 26:3992–4003. 10.1038/s41380-020-0663-2 32015467 PMC8855973

[5] Kopasz M Loessl B Hornyak M Riemann D Nissen C Piosczyk H Sleep and memory in healthy children and adolescents—a critical review. Sleep Med Rev. (2010) 14(3):167–77. 10.1016/j.smrv.2009.10.006 20093053

[6] Gomez RL Newman-Smith KC Breslin JH Bootzin RR. Learning, memory, and sleep in children. Sleep Med Clin. (2011) 6(1):45–57. 10.1016/j.jsmc.2010.12.002

[7] Quist JS Sjödin A Chaput J Hjorth MF. Sleep and cardiometabolic risk in children and adolescents. Sleep Med Rev. (2015) 29:76–100. 10.1016/j.smrv.2015.09.001 26683701

[8] Gerber L. Sleep deprivation in children. Nursing (Brux). (2014) 44(4):50–4. 10.1097/01.nurse.0000441881.87748.90 24646580

[9] Matricciani L Olds T Petkov J. In search of lost sleep: secular trends in the sleep time of school-aged children and adolescents. Sleep Med Rev. (2012) 16(3):203–11. 10.1016/j.smrv.2011.03.005 21612957

[10] Wheaton AG Claussen AH. Short sleep duration among infants, children, and adolescents aged 4 months–17 years — United States, 2016–2018. MMWR Morb Mortal Wkly Rep. (2021) 70:1315–21. 10.15585/mmwr.mm7038a1 34555000 PMC8459893

[11] Li L Zhang S Huang Y Chen K. Sleep duration and obesity in children: a systematic review and meta-analysis of prospective cohort studies. J Paediatr Child Health. (2017) 53(4):378–85. 10.1111/jpc.13434 28073179

[12] Miller MA Kruisbrink M Wallace J Ji C Cappuccio FP. Sleep duration and incidence of obesity in infants, children, and adolescents: a systematic review and meta-analysis of prospective studies. Sleep. (2018) 41(4):zsy018. 10.1093/sleep/zsy018 29401314

[13] Morales-Muñoz I Upthegrove R Lawrence K Thayakaran R Kooij S Gregory AM The role of inflammation in the prospective associations between early childhood sleep problems and ADHD at 10 years: findings from a UK birth cohort study. J Child Psychol Psychiatry. (2023) 64(6):930–40. 10.1111/jcpp.13755 36597271 PMC10952536

[14] Morales-Muñoz I Gregory AM. Sleep and mental health problems in children and adolescents. Sleep Med Clin. (2023) 18(2):245–54. 10.1016/j.jsmc.2023.01.006 37120167

[15] Chaput J Gray CE Poitras VJ Carson V Gruber R Olds T Systematic review of the relationships between sleep duration and health indicators in school-aged children and youth. Appl Physiol Nutr Metab. (2016) 41(6 (Suppl. 3)):S266–82. 10.1139/apnm-2015-0627 27306433

[16] Galland BC Short MA Terrill P Rigney G Haszard JJ Coussens S Establishing normal values for pediatric nighttime sleep measured by actigraphy: a systematic review and meta-analysis. Sleep. (2018) 41(4):1–16. 10.1093/sleep/zsy017 29590464

[17] Meltzer LJ Montgomery-Downs HE Insana SP Walsh CM. Use of actigraphy for assessment in pediatric sleep research. Sleep Med Rev. (2012) 16(5):463–75. 10.1016/j.smrv.2011.10.002 22424706 PMC3445439

[18] Meltzer LJ Avis KT Biggs S Reynolds AC Crabtree VM Bevans KB. The children’s report of sleep patterns (CRSP): a self-report measure of sleep for school-aged children. J Clin Sleep Med. (2013) 09(03):235–45. 10.5664/jcsm.2486 PMC3578690 23493949

[19] Meredith-Jones KA Haszard JJ Graham-DeMello A Campbell A Stewart T Galland BC Validation of actigraphy sleep metrics in children aged 8 to 16 years: considerations for device type, placement and algorithms. Int J Behav Nutr Phys Act. (2024) 21(1):40. 10.1186/s12966-024-01590-x 38627708 PMC11020269

[20] Berry RB. Hypersomnias of central origin. In: Goolsby J Pritchard J, editors. Elsevier EBooks. Amsterdam: Elsevier (2011). p. 451–79. 10.1016/b978-1-4377-0326-9.00024-5

[21] Pacheco D Rehman A. Sleep Latency. Seattle, WA: Sleep Foundation (2023). Available online at: https://www.sleepfoundation.org/how-sleep-works/sleep-latency#references-80145

[22] Shrivastava D Jung S Saadat M Sirohi R Crewson K. How to interpret the results of a sleep study. J Community Hosp Intern Med Perspect. (2014) 4(5):24983. 10.3402/jchimp.v4.24983 25432643 PMC4246141

[23] Suni E Vyas N. Wakefulness After Sleep Onset. Seattle, WA: Sleep Foundation (2023). Available online at: https://www.sleepfoundation.org/sleep-studies/wakefulness-after-sleep-onset

[24] O’Brien LM. The neurocognitive effects of sleep disruption in children and adolescents. Sleep Med Clin. (2009) 18(4):813–23. 10.1016/j.jsmc.2010.12.007 19836689

[25] Touchette É Petit D Paquet J Boivin M Japel C Tremblay RE Factors associated with fragmented sleep at night across early childhood. Arch Pediatr Adolesc Med. (2005) 159(3):242. 10.1001/archpedi.159.3.242 15753267

[26] Child and Adolescent Health Measurement Initiative. 2022-2023 National Survey of Children’s Health (NSCH) Data Query. Baltimore, MD: Data Resource Center for Child and Adolescent Health supported by the U.S. Department of Health and Human Services, Health Resources and Services Administration (HRSA), Maternal and Child Health Bureau (MCHB) (2024). Available online at: https://nschdata.org/browse/survey/results?q=11292&g=1148&r=41

[27] United Health Foundation. 2024 Health of Women and Children Report. Eden Prairie, MN: The United Health Foundation (2024). Available online at: AmericasHealthRankings.org; https://assets.americashealthrankings.org/app/uploads/ahr_2024hwc_statesummaries-all.pdf

[28] Wheaton AG Claussen AH. FastStats: Sleep in Children. Atlanta, GA: Sleep (2024). Available online at: https://www.cdc.gov/sleep/data-research/facts-stats/children-sleep-facts-and-stats.html

[29] McDowall PS Galland BC Campbell AJ Elder DE. Parent knowledge of children’s sleep: a systematic review. Sleep Med Rev. (2017) 31:39–47. 10.1016/j.smrv.2016.01.002 26899741

[30] Dayyat N Spruyt N Molfese N Gozal D. Sleep estimates in children: parental versus actigraphic assessments. Nat Sci Sleep. (2011) 3:115–23. 10.2147/nss.s25676 23616722 PMC3630966

[31] Mazza S Bastuji H Rey AE. Objective and subjective assessments of sleep in children: comparison of actigraphy, sleep diary completed by children and parents’ estimation. Front Psychiatry. (2020) 11:495. 10.3389/fpsyt.2020.00495 32587532 PMC7297917

[32] Werner H Molinari L Guyer C Jenni OG. Agreement rates between actigraphy, diary, and questionnaire for children’s sleep patterns. Arch Pediatr Adolesc Med. (2008) 162(4):350–8. 10.1001/archpedi.162.4.350 18391144

[33] Short MA Gradisar M Lack LC Wright HR Chatburn A. Estimating adolescent sleep patterns: parent reports versus adolescent self-report surveys, sleep diaries, and actigraphy. Nat Sci Sleep. (2013) 5:23–6. 10.2147/NSS.S38369 23620690 PMC3630985

[34] Loredo JS Soler X Bardwell W Ancoli-Israel S Dimsdale JE Palinkas LA. Sleep health in U.S. Hispanic population. Sleep. (2010) 33(7):962–7. 10.1093/sleep/33.7.962 20614856 PMC2894438

[35] Cheng TL Goodman E Bogue CW Chien AT Dean JM Kharbanda AB Race, ethnicity, and socioeconomic status in research on child health. Pediatrics. (2015) 135(1):e225–37. 10.1542/peds.2014-3109 25548336 PMC9923597

[36] Flores G Fuentes-Afflick E Barbot O Carter-Pokras O Claudio L Lara M The health of Latino children: urgent priorities, unanswered questions, and a research agenda. JAMA. (2002) 288(1):82–90. 10.1001/jama.288.1.82 12090866

[37] Guglielmo D Gazmararian JA Chung J Rogers AE Hale L. Racial/ethnic sleep disparities in US school-aged children and adolescents: a review of the literature. Sleep Health. (2017) 4(1):68–80. 10.1016/j.sleh.2017.09.005 29332684 PMC5771439

[38] Wang Y Zhao Z Zhang Y Yan J Zhang M Jelsma E Race, ethnicity, and sleep in US children. JAMA Network Open. (2024) 7(12):e2449861. 10.1001/jamanetworkopen.2024.49861 39656455 PMC11632548

[39] Schmied EA Full KM Lin SF Gregorio-Pascual P Ayala GX. Sleep health among U.S. Hispanic/Latinx children: an examination of correlates of meeting sleep duration recommendations. Sleep Health. (2022) 8(6):615–9. 10.1016/j.sleh.2022.07.002 36055935

[40] Wallace AL Aguinaldo L Thomas ML McCarthy MJ Meruelo AD. Preliminary findings on caffeine intake, screen time, social factors, and psychological well-being: their impact on chronotype and sleep health in Hispanic adolescents. Sleep Adv. (2025) 6(2):zpaf019. 10.1093/sleepadvances/zpaf019 40303380 PMC12038348

[41] Blanco E Hyde ET Martinez SM. Assessing sleep behaviors in Latino children and adolescents: what is known, what are we missing, and how do we move forward? Curr Opin Pediatr. (2024) 36(1):17–22. 10.1097/MOP.0000000000001306 37933679 PMC10825877

[42] U.S. Census Bureau. Rhode Island’s Population Grew 4.3% Last Decade. Suitland, MD: United States Census Bureau (2023). Available online at: Census.gov; https://www.census.gov/library/stories/state-by-state/rhode-island-population-change-between-census-decade.html#race-ethnicity

[43] Grigsby-Toussaint DS Shin JC Acevedo AR Kemball-Cook W Story D Katz A Project G-SPACE: protocol for exploring the influence of green space on sleep and mental health among children. BMC Pediatr. (2024) 24(1):783. 10.1186/s12887-024-05247-3 39614236 PMC11606230

[44] Owens JA Spirito A McGuinn M. The children’s sleep habits questionnaire (CSHQ): psychometric properties of a survey instrument for school-aged children. Sleep. (2000) 23(8):1043–51. 10.1037/t33022-000 11145319

[45] Paruthi S Brooks LJ D’Ambrosio C Hall WA Kotagal S Lloyd RM Recommended amount of sleep for pediatric populations: a consensus statement of the American academy of sleep medicine. J Clin Sleep Med. (2016) 12(6):785–6. 10.5664/jcsm.5866 27250809 PMC4877308

[46] Combs D Goodwin JL Quan SF Morgan WJ Parthasarathy S. Longitudinal differences in sleep duration in hispanic and Caucasian children. Sleep Med. (2016) 18:61–6. 10.1016/j.sleep.2015.06.008 26299467 PMC4548806

[47] Schachter FF Fuchs ML Bijur PE Stone RK. Cosleeping and sleep problems in Hispanic-American urban young children. Pediatrics. (1989) 84(3):522–30. 2788867

[48] Hoyniak CP Bates JE Camacho MC McQuillan ME Whalen DJ Staples AD The physical home environment and sleep: what matters most for sleep in early childhood. J Fam Psychol. (2022) 36(5):757–69. 10.1037/fam0000977 35266772 PMC9747092

[49] Cohn D Menasce Horowitz J Minkin R Fry R Hurst K. 1. the Demographics of Multigenerational Households. Washington, DC: Pew Research Center (2022). Available online at: https://www.pewresearch.org/social-trends/2022/03/24/the-demographics-of-multigenerational-households/#:∼:text=Among%20major%20racial%20and%20ethnic,of%20those%20who%20are%20White

[50] Liu X Liu L Wang R. Bed sharing, sleep habits, and sleep problems among Chinese school-aged children. Sleep. (2003) 26(7):839–44. 10.1093/sleep/26.7.839 14655917

[51] Martin JL Hakim AD. Wrist actigraphy. Chest. (2011) 139(6):1514–27. 10.1378/chest.10-1872 21652563 PMC3109647

[52] Hirshkowitz M Whiton K Albert SM Alessi C Bruni O DonCarlos L National sleep foundation’s updated sleep duration recommendations: final report. Sleep Health. (2015) 1(4):233–43. 10.1016/j.sleh.2015.10.004 29073398

[53] RStudio Team. RStudio: Integrated Development for R. Boston, MA: RStudio, PBC (2020). Available online at: http://www.rstudio.com/

